# Do Fallers and Nonfallers Equally Benefit from Balance Specific Exercise Program? A Pilot Study

**DOI:** 10.1155/2013/753298

**Published:** 2013-10-21

**Authors:** Darja Rugelj, Marija Tomšič, France Sevšek

**Affiliations:** Faculty of Health Sciences, University of Ljubljana, Zdravstvena pot 5, 1000 Ljubljana, Slovenia

## Abstract

The purpose of the study was to determine the sample size that would allow broad generalizability of the results. To investigate the differences in the responsiveness of fallers and nonfallers to a multicomponent functional balance specific program, 23 participating subjects (70.1 ± 6.6 years) were divided into nonfallers group (13) and fallers group (10). The components of the balance specific program were (1) changing of the center of gravity (CoG) in the vertical direction, (2) shifting of the CoG to the border of stability, (3) rotation of the head and body about the vertical axis, (4) standing and walking on soft surface, and (5) walking over obstacles or on a narrow path. At the end of eight months of the training program, there was no significant difference between the two groups regarding postural sway. The total center of pressure path length was used as the principal outcome measure for the sample size calculation. Based on these results the a priori sample size calculation yielded the estimate of 110 subjects required to be enrolled in order to get 20 subjects in fallers and 30 subjects in nonfallers group for the 80% power to detect the results as significant.

## 1. Introduction

The phenomenon of accidental falls has been associated with multiple causes, divided into intrinsic and extrinsic risk factors [1]. The extrinsic factors result from persons' environment such as thick carpeting, improper footwear, slippery surfaces, and poor illumination. On the other hand, the most often identified intrinsic factors are those related to the medication use, syncope, postural instability, loss of balance, visual impairment, muscle weakness, and sensomotor deficit [1]. Most often falls among elderly community-dwelling subjects occur while walking on level or uneven surfaces [2], and the majority of them occur during dual tasking while walking [3]. Accidental falls in the advanced age represent a serious health problem. From the available data for the year 2010, over 60% of total hospital treatments in Slovenia were caused by falls [4].

Strategies for fall prevention should address both the intrinsic and extrinsic risk factors [5]. In the intrinsic risk factors domain, balance is an important risk factor for the falls among the elderly frail individuals as well as among the community-dwelling ones [6]. Therefore, a number of training protocols have been proposed to enhance or maintain balance into advanced age. The results of the reviews indicate that there is a weak evidence that some exercise types are moderately effective in improving balance [7]. For the community-dwelling older adults a progressive exercise program that focuses on moderate to high intensity balance exercise appears to be one of the most effective interventions to prevent falls [8]. The emphasis is on functional balance specific program where subjects practice activities similar to those of their daily life. This kind of program has been shown to be effective for a group of nursing home residents [9] as well as for the community dwelling ones [10–12].

Differences between fallers and nonfallers in their functional capabilities are numerous as shown by the results of different balance tests. Thus, the required time is significantly longer to perform the four-square step test [13], as well as the Timed up and Go Test [14]. The fallers' scores are lower in Berg balance scale and Activities-specific Balance Confidence (ABC) scale, and there is a larger high-frequency component of the postural sway in fallers as compared to nonfallers [1]. Mediolateral sway has also been shown to be a reliable predictor of falls [15]. Fallers have also a significantly longer reaction time [1] and increased fear of falling [16], and their gait speed has been found to be slower under both normal [17] and dual-task conditions [18].

Physiological factors that are correlated with falls are diminished proprioception in the lower limbs and slower reaction time. These are the variables that significantly discriminated between subjects who experienced multiple falls and the ones who experienced a single fall or none at all [19]. Physiological differences between fallers and nonfallers may have a potential effect on their capability to differently respond to certain training strategies. The research question is whether there are differences in the responsiveness of fallers and nonfallers to a multicomponent balance specific exercise program. This is important in the process of clinical decision making. In case the fallers and nonfallers respond equally to treatment, no specifically individualized program is required for them. The purpose of this pilot study was to determine the feasibility of the study and to calculate the appropriate sample size based on determined clinical and stabilometric output measures.

Our working hypothesis is that older community dwelling adults who had experienced an unexpected fall will (1) benefit equally from the balance specific program as the nonfallers, and therefore (2) no specific individualized exercise program is required for the faller group.

## 2. Methods

### 2.1. Participants

Residents of the Ljubljana town area were invited to participate in this study. The program was presented in pensioners' clubs and in day centers; additionally, invitations were published as leaflets and distributed in clubs and day centers; The study was approved by the Slovenian Medical Ethic Committee. At the beginning of the study subjects signed a written consent. The inclusion criteria for the final analysis of the collected data were age above 65 years, score of 46 points as obtained by the Berg balance scale (BBS), no neurological conditions, and a 75-percent adherence rate. Thus, out of 55 subjects, 23 subjects (21 female and 2 male) met the inclusion criteria and were included in the final analysis and had the mean age of 70.1 ± 6.6 years, body mass of 67.9 ± 12.1 kg, and body height of 162 ± 6.6 cm.

For the analysis the subjects who regularly attended the balance specific program were divided into two groups according to the history of falls: the nonfallers group which consisted of 13 subjects who did not experience any fall in the past 8 months and the fallers group which consisted of 10 subjects who had experienced at least one fall in this period. A fall was defined as an unintentional coming to rest on the ground or on any lower level. Six falls occurred outdoors (on a street or path, and one subject fell while skiing), whereas three of them happened indoors. There is no data for the fall location of one subject. As a consequence of the fall, five subjects visited their medical doctor, but no falls resulted in hospitalization.

### 2.2. Functional Performances Measures

The levels of functional fitness and the level of balance prior to the training program were assessed with different functional tests. For balance, the Berg balance scale was used which consists of 14 functional activities graded on a scale from 0 to 4. It is valid [20] and reliable [21]. Motor performances of the lower extremities were tested by the Timed stance on toes test [22] whereas the hand grip strength was estimated using a hydraulic hand dynamometer (Jamar, Lafayette Instruments, USA).

Additionally, the dynamic aspect of balance and its integration into gait were assessed by evaluating the performance at the stance on a narrow base (Sharpened Romberg test), four-square step test [13], and Timed 10 m walk test. For the Sharpened Romberg test, the maximum time was fixed to 60 seconds while the subjects were standing with their eyes opened and closed. The four-square step test was used to assess subjects' agility, weight transfer, and change of direction. It is reliable and valid clinical tool where an additional cognitive component is included since the subjects are required to remember the sequences of the test: at the end of the first cycle they need to change the direction and repeat stepping in the reversed order. At the Timed 10 m walk test, the subjects were required to walk in a corridor that was marked at distances of 0, 2, and 10 m. The first two-meter distance allowed the subjects to develop their walking speed whereas the next ten-meter walks were timed by the therapist who stood at the midpoint. This test has been shown to be a reliable measure of functional mobility [23].

### 2.3. Modified Sensory Organization Test on Force Platform

The modified sensory organization test was used as the principal outcome measure to assess the effectiveness of balance specific exercise program. It is a well-established [24] clinical tool for the assessment of the relative contribution of proprioceptive, vestibular, and vision systems to postural integration. The measurements of postural sway by stabilometry were done in four different conditions: standing on hard surface and on the Airex mat (40 × 48 × 6 cm) with eyes opened and closed using Kistler 9286 AA (Winthertur, Switzerland) force platform with 50 Hz sampling rate. The subjects were instructed to stand barefoot on the force platform as still as possible for 60 seconds with arms being at their sides and the feet close together. For each subject a set of four tests was done.

Raw data were collected by BioWare program and then uploaded to a server with Linux operating system where a specially developed software [25] was used for the analysis. The data were smoothed by Gaussian filtering, and the time and frequency distributions were calculated and plotted. The standard stabilometric parameters, such as the mediolateral and anteroposterior center of pressure (CoP) path lengths and total path length, were determined. Additionally, the outlines of the measured stabilometric data were expressed in terms of the first 25 Fourier coefficients to evaluate the shapes and areas of the region visited by the center of pressure during quiet standing [26]. For the purpose of present analysis four sway variables were chosen: total path length of the CoP during 60 s measurement interval, mediolateral and anteroposterior sway expressed as mediolateral and anteroposterior CoP path lengths, and the sway area which was calculated from the first 25 Fourier coefficients of the area outline.

### 2.4. Statistical Analysis

The Statistical Package for Social Sciences (SPSS 20, SPSS Inc., Chicago, IL USA) was used for statistical analysis. All dependent variables were visually inspected for normality which was found in all cases. An independent sample *t*-test was used to assess the two groups before the beginning of training for age, body mass, functional balance, strength, and the sensory organization test on force platform and to assess a response to intervention expressed as gain (difference between initial and final results). Further, a Pearson correlation coefficient was used to determine the significance of the relationships between variables. Afterwards to determine whether the groups differed in their improvement during the study period in postural steadiness in different sensory conditions, a two-way repeated measures analysis of variance (ANOVA) was performed. The significance level for all tests was set to *P* < 0.05.

Sample size was calculated with G*Power 3.1.7 [27] for the main outcome variable. To obtain the main outcome variable for the sample size calculation, the correlation coefficients were calculated between functional performance measures and variables of postural sway as obtained by modified sensory organization test on force platform.

### 2.5. Exercise Program

The training protocol lasted 8 months with two sessions per week, each of them consisting of two parts: the first 30 minutes were devoted to general exercises (e.g., activation of major muscle groups of the upper and lower extremities, as well as trunk muscles and the range of motion exercises), followed by 30 minutes of balance specific training.

### 2.6. Basic Components of Balance Specific Exercise Program

The balance specific activities were designed to emphasize different features of balance function. The components were (1) changing of the center of gravity (CoG) position in the vertical direction, (2) shifting of the CoG to the border of stability, (3) rotation of the head and body about the vertical axis, (4) standing and walking on soft supporting surface, and (5) walking over obstacles or on a narrow path.

The first component was changing of the CoG position in the vertical direction. This component is required for transitions between different stable positions such as standing up and sitting down as well as for ascending and descending stairs. Stair climbing and transitions between positions are activities during which high percentage of indoor falls occur [16]. For the shifting of the CoG in vertical direction, a certain amount of strength of the thigh muscles is required. Additionally for stair climbing a certain amount of aerobic capacity is required as well as the perception of depth. All of these elements (rising of CoG, aerobic demand, and depth perception) are included in the exercises on steppers.

The second component of the balance specific program consisted of shifting the center of gravity to the border of stability. This skill is required when reaching beyond the arm length and is known to decrease in elderly subjects [24].

The third component required rotation of the head and body about the vertical axis. This skill is necessary for avoiding obstacles and while looking over the shoulder where head movement is followed by whole body axial rotation. This movement elicits the vestibuloocular response that is responsible for gaze stabilization and is closely related to postural control [28].

The fourth component consisted of the activities done while standing on a soft supporting surface. This skill is for instance required for walking on thick carpeting or during certain outdoor activities such as walking in meadows or woods. Exercises on soft and compliant surfaces facilitate postural stabilization with a decreased amount and changed quality of afferent flow from the proprioceptive systems. On a compliant surface, the pressure on the sole is distributed over a larger area [29]. The other effect imposed by the compliant surface is its elasticity which requires constant adjustments to moving surface, and adjustments of the intersegmental positions of the body [30]. Training on a movable supporting surface has been reported to decrease the destabilizing effect of movable surfaces [31] and improves intersegmental coordination [10].

The fifth component consisted of walking over obstacles or on narrow surfaces. This kind of walking, while subjects put their legs in front of each other, requires more control in the hip region and enhances the training of hip strategy that has been reported as a prevailing balance movement strategy in elderly subjects [32].

The balance specific program was organized as a circuit training on typically three stations as described in detail elsewhere [12]. The first one was training on a compliant surface and was used in all training sessions; the other two stations were chosen between obstacle avoidance station organized as polygon, steppers, and seated activities on gym balls. The last activity was always a dance, mostly a group folk dance. Subjects spent about 8 minutes on each of the three stations. On each station, one or two assistants were present at all times to provide sufficient security.

## 3. Results

### 3.1. Results of the Pilot Study

#### 3.1.1. Pretraining Assessments

In the first stage of the data analysis, the results of the initial assessments, before the training protocol started, were compared. They showed that the fallers and nonfallers groups did not statistically significantly differ in age, body mass and height, hand grip, and calf muscles strength (with *P* values ranging from 0.44 to 0.783). The average hand grip strength is presented in Table 1 for women and separately for the two male participants. The initial balance tests were similar in both groups (Table 1) except for the fast walking speed that statistically significantly differed (*P* = 0.028) at the initial assessment between the fallers, and nonfallers group with the former being faster.

#### 3.1.2. Post-Training Assessment

*Sensory Organization Test: Hard Surface*. For a 2 × 2 (postural sway variable × group) between-subjects repeated measures ANOVA for eyes opened condition there was no significant main effect for all variables indicating that there was no effect of balance training on CoP measures with *P* values ranging from the highest value of 0.877 for mediolateral path length to *P* = 0.245 for sway area. However, there was a significant interaction for the total path length (*F* = 5.856, *P* = 0.025), mediolateral sway (*F* = 4.467, *P* = 0.047), and anteroposterior sway (*F* = 6.982, *P* = 0.015), indicating that there is an effect of group. The values expressed as means and standard deviations are given in Table 2.

When measured with eyes closed there was a significant main effect for the total path length (*F* = 4.519, *P* = 0.046), and mediolateral sway (*F* = 4.817, *P* = 0.040) indicating that there is an effect of balance training on CoP movements. The interaction between the groups was not significant for the total path length (*F* = 3.285, *P* = 0.084) and mediolateral sway (*F* = 2.725, *P* = 0.114) indicating that there are no differences between the two groups. There was no significant main effect for anteroposterior sway (*F* = 3.239, *P* = 0.086) and sway area (*F* = 0.48, *P* = 0.829).

The detailed results (average values and their standard deviations) for the four variables calculated for both groups while standing on hard surface are presented in the Table 2.

*Sensory Organization Test: Compliant Surface*. For a 2 × 2 (postural sway variable × group) between-subjects repeated measures ANOVA for eyes opened condition there was a significant main effect for total path length (*F* = 8.436, *P* = 0.008), mediolateral sway (*F* = 6.783, *P* = 0.017), and sway area (*F* = 11.841, *P* = 0.002), indicating that there is an effect of balance training on the measures of postural sway. The interaction was not significant: total path length (*F* = 0.253, *P* = 0.620), mediolateral sway (*F* = 0.071, *P* = 0.793), and sway area (*F* = 1.656, *P* = 0.212), indicating that there is no effect of group. There was no significant main effect for anteroposterior sway (*F* = 3.478, *P* = 0.076) indicating that there is no effect of balance training on anteroposterior sway The precise values expressed as means and standard deviations are noted in Table 3.

When measured with eyes closed, there was a significant main effect for mediolateral sway (*F* = 7.274, *P* = 0.018) and sway area (*F* = 5.549, *P* = 0.032), indicating that there is an effect of balance training on the measures of postural sway. However, the interaction was not significant for mediolateral sway (*F* = 3.811, *P* = 0.069) and sway area (*F* = 3.200, *P* = 0.093) indicating that there is no group effect. No significant main effect was found for total path length (*F* = 3.105, *P* = 0.097) and anteroposterior sway (*F* = 0.595, *P* = 0.452). The values expressed as means and standard deviations are noted in Table 3.

### 3.2. Sample Size Calculation

The a priori sample size was calculated on the basis of the results from the present pilot study.

To obtain the main outcome variable for the sample size calculation, the differences were calculated between the pre- and posttraining values of two CoP variables of the sensory organization test. The initial functional levels of the subjects' balance were taken into consideration by determining the correlations of these differences with the functional performances measures. Significant correlation coefficient was obtained between BBS and two CoP variables. The correlation coefficients of the CoP variables for standing on hard surface with eyes opened were *r* = 0.478 (*P* = 0.05) for the total path length and *r* = 0.520 (*P* = 0.05) for the mediolateral path length, whereas, for standing on hard surface with eyes closed, they were *r* = 0.653 (*P* = 0.01) and *r* = 0.661 (*P* = 0.01) for the total and mediolateral path lengths, respecitvely. The correlation coefficients calculated for standing on compliant surface with eyes closed and eyes opened were low and not significant.

Thus, the variables that correlated most with the functional balance state of the subjects as measured with BBS were the total path length of CoP and mediolateral sway. As these two variables are interrelated, the total path length of the CoP was chosen for the a priori sample size calculation using the average value of the differences between the pre- and posttraining results and its standard deviation. The analysis with G*Power software showed that at least 20 subjects per group are needed to provide 80% power to detect the results as significant at alpha level 5%.

**Figure 1:**
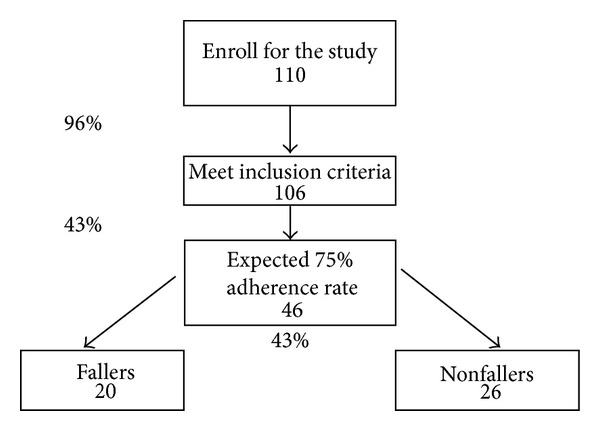
Flow chart of subjects needed for recruitment to obtain sufficient sample size taking into consideration adherence to the training sessions.

Therefore taking into consideration that there are 43% of fallers expected in the study group as determined by this study, further considering the expected adherence rate bee 43% and that 96% of the enrolled subjects will meet the inclusion criteria, it can be concluded that at least 110 subjects should be recruited to achieve the sufficient statistical power of the stabilometric tests. Thus, 106 subjects are expected to meet the inclusion criteria, resulting in 46 subjects who are likely to regularly attend the balance specific exercise program and subsequently resulting in 20 subjects in the fallers and 26 subjects in the nonfallers group (Figure 1).

## 4. Discussion

The purpose of the present pilot study was to obtain the sample size to enable broad generalizability of the results to test the hypothesis that subjects who had experienced at least one accidental fall in the past year have equal potential to benefit from the balance specific program as compared to nonfallers. For the chosen main outcome measure (the CoP path length) the number of 110 subjects was determined. Based on the results of the present pilot study, we could have in principle confirmed the hypothesis since we found no significant difference between the two groups at the end of eight months of the exercise program regarding measures of postural stability (total path length, mediolateral and anteroposterior sway, and sway area). However, the sample size prevents us from making this conclusion. The choice of the outcome measure was carefully tested. The total path length of CoP was chosen as the main outcome measure for the sample size calculation. This was the variable that significantly correlated with the BBS which has become a gold standard in clinical balance measurements and has a well-established validity [20, 21]. Additionally, two functional tests, a four-square test and Timed 10 m walk test, were proven to be needed to cover the dynamic aspects of balance.

It has been reported that regardless of the degree of fall risk at the enrollment time the subjects responded to the intervention aimed at fall prevention or decrease of fall risk factors [33]; however, it has not been reported whether fallers and nonfallers responded equally to the same type and amount of training. The subjects under study are still highly functional and are not suspected to have an increased fall risk due to balance as indicated by Berg balance score where 46 points and below are considered as the cutoff value for the increased fall risk [1]. Nevertheless, also the subjects who still have preserved balance, according to our data, had experienced, a fall, and only one of the ten falls was associated with a recreational activity. Additionally, subjects with an experience of a fall seem to be more likely to attend regularly to the balance specific exercise program since the ratio between fallers and nonfallers differed to those in general population [6] since in our study 43% of participants experienced a fall.

No initial difference in balance performance between the participants of both groups was found in the stabilometric and functional tests in the present study. The only initial difference was in walking speed of the fallers group that was for fast walking significantly slower. The results of the functional tests are in agreement with Melzer et al. [34] who reported no differences between fallers and nonfallers in a similar highly functional group for BBS score and Timed Up and Go Test. However, the reported stabilometric results showed larger mediolateral sway range in fallers as compared to nonfallers under eyes closed condition on hard supporting surface [34], while there was no difference between the two currently reported groups in the mediolateral sway in neither of the four conditions of the modified sensory organization test. This difference between our study and the previously reported one could be attributed to the definition of fallers: our study defined a faller as someone who had incidentally fallen at least once in the past 8 months whereas in the study of Melzer et al. [34] a single fall in the past year was considered as a random event that did not reflect a balance disorder [15, 35], and only subjects with two or more falls were considered as fallers. However, the reported study was prospective, and we have now knowledge as to what extent a subject was a recurrent faller or not. For the future study, a combination of retrospective as well as prospective data on fall status will be required to differentiate between fallers and those whose fall could be considered as a random event. Regardless of the number of falls the outdoor fallers were found to have very good overall health [16].

The mediolateral sway as an indicator of stability revealed no differences between the two studied groups. In both groups the same decrease was obtained for the mediolateral sway on a compliant surface. With age a shift towards hip strategy for balance reactions is apparent [32]; therefore, activities that have a potential to preserve and enhance motor control and coordination in the hip are likely to influence the balance and have potential to decrease the risk for falling. Our results indicate that the described balance specific program can influence the hip control equally in the subjects who had experienced falls as well as in those who had not. Mediolateral stability has improved on compliant surface in fallers as well as in nonfallers. The control of lateral stability appears to be the most important variable to discriminate between fallers and nonfallers [15, 34].

Mediolateral control is required also in a variety of functional activities such as changing direction, stepping sideways, backwards, and stepping on and off stairs and for the tasks related to the obstacle avoidance. Decreased efficacy of this function is related to the increased fall risk [13]. The specific training for the obstacle avoidance has been reported to be effective in fall prevention programs of community dwelling elderly subjects [36]. Other types of training programs, such as step aerobics, were reported to be efficient for improving one leg stance and walking on narrow basis [37]. To test functional aspect of balance specific exercise program, the four-square step test should be introduced as an outcome measure. Four-square step test is a measure of mediolateral control, weight transfer, change of direction, and agility. Subjects in both groups of the reported study performed very well in the four-square step test, much better indeed as expected of a fall prone group as reported by Dite and Temple [13].

Training on a compliant surface is an important part of the functional balance training program, and fallers and nonfallers responded equally and significantly to it. Present results suggest that during the training both groups to the same extant learned to respond to instability of the supporting compliant surface fast enough to maintain postural stability. The mediolateral sway, total path length, and sway area on a compliant surface decreased in both groups of participants. The elasticity of the supporting surface results in additional body movement which requires constant adjustments of the relative positions of the body segments to keep the center of gravity over the base of support [30]. The consequence of these constant adjustments is increased afferent flow from the muscle and joint receptors [38]. Therefore, our results support the enhancement of the proprioceptive acuity, which has been related to the training on unstable surfaces [38]. Previous reports of training on compliant and moving surfaces are however conflicting; some authors indicate that such a sensory-specific training reduces the influence of the mechanical destabilization on body balance [31], and reduces postural sway [12]; training on the wobble board has been shown to significantly decrease the amount of ankle movements [39] and improves the intermuscular coordination [10] while others report no effect on the CoP stability [40].

Walking speed was the only variable that differed in the initial assessment between the two groups. It has been previously reported that fallers walk more slowly as compared to the nonfallers [17]. Walking is one of the functions that allows elderly subjects to stay independent and is closely related to balance [41]. With the improvement of balance the gait speed improved although the gait speed was not part of the training in a group of nursing home residents [9] as well as among community-dwelling subjects [12]. Therefore, gait speed should be also used as a measure of the transfer of the training program to functional activities.

The balance specific program is designed to be translated to daily living activities. The intensity of the training is low; therefore, it is not expected that changes be attributed to increased functional load [42], and it is more likely that the observed improvement is a result of motor learning process. The process can be categorized as implicit learning which is defined as a nonconscious form of learning characterized by behavioral improvement [43]. Not only can the new postural synergies be learned but also the newly acquired synergies influence the existing ones [44]. The less stable are the preexisting coordination patterns, the more change can be induced as a consequence of learning [44].

It may be concluded that multicomponent, balance specific program that includes (1) changing of the CoG in the vertical direction, (2) shifting of the CoP to the border of supporting surface, (3) rotation of the head and body about the vertical axis, (4) standing on soft supporting surface, and (5) walking over obstacles or on a narrow path has a potential to increase postural steadiness in active community-dwelling subjects; and it has yet to be determined whether or not this is the case for faller and nonfallers equally. Based on the results of this preliminary study we cold calculate an appropriate sample site to ensure sufficient statistical power to allow generalizability of the acquired results regarding whether or not multicomponent balance specific program is optimal in its ability to enhance balance control in subjects who had experienced a sudden fall as well as in those subjects who had not experienced it.

## Figures and Tables

**Table 1:** Descriptive statistics for the two groups and their initial functional measurements for strength, balance, and gait speed tests.

	Nonfallers group ± SD	Fallers group	Difference between groups (*P*)
Age (years)	69.5 ± 6.5	71.7 ± 6.7	0.44
Height (cm)	161.1 ± 5.9	158.9 ± 7.9	0.458
Weight (kg)	69 ± 9.9	67.6 ± 14.8	0.783
Berg balance scale (pts.)	54.5 ± 2	54.7 ± 1.6	0.776
Standing on the toes (s)	57.6 ± 8.3	48.9 ± 15.2	0.108
Hand grip strength: female			0.284
Left hand (kg)	26.2 ± 5.9	23 ± 6.6	
Right hand (kg)	28.2 ± 7.9	26.8 ± 7.9	0.654
Hand grip strength: male (two subjects, 1 and 2)		—	
Left hand (kg)	39 and 42		
Right hand (kg)	40 and 45	—	
Four-square step test (s)	9.24 ± 2.1	9.5 ± 1.4	0.794
10 m walk test (s)	6.7 ± 1.6	8.1 ± 0.9	**0.028**
Sharpened Romberg test: eyes open (seconds)	54.2 ± 15.7	50.8 ± 19.2	0.656
Sharpened Romberg test: eyes closed (seconds)	20.7 ± 22	21 ± 19.4	0.973
Adherence to training (times)	39.1 ± 6.5	39 ± 6.8	0.978

Significant differences between the two groups are indicated in bold.

**Table 2:** Average values and their standard deviations for the four CoP variables calculated for both groups while standing on hard surface.

Group	Non-fallers eyes opened	Fallers eyes opened	Non-fallers eyes closed	Fallers eyes closed
*Variable *				
Total path length (cm)				
Before training	77.45 ± 22.52	80.00 ± 31.54	124.31 ± 72.56	109.81 ± 39.98
After training	90.99 ± 32.50	68.79 ± 22.99	156.51 ± 115.29	112.38 ± 38.02
Mediolateral path (cm)				
Before training	54.5 ± 13.6	58.2 ± 26.7	81.8 ± 32.7	81.9 ± 31.7
After training	65 ± 19.4	49.2 ± 18	107.5 ± 62.7	85.6 ± 30.7
Anteroposterior path (cm)				
Before training	43.24 ± 19.64	43.03 ± 15.3	74.23 ± 61.83	56.58 ± 22.7
After training	50.45 ± 23.72	37.92 ± 12.14	90.65 ± 84.32	55.62 ± 19.31
Sway area (cm^2^)				
Before training	4.9 ± 2.4	6.45 ± 5.6	7.2 ± 4.6	7.5 ± 9.1
After training	4.9 ± 1.7	4.35 ± 3.0	8.9 ± 7.3	6.3 ± 5.4

**Table 3:** Average values and their standard deviations for the four variables calculated for both groups while standing on a compliant surface.

Group	Non-fallers eyes open	Fallers eyes open	Non-fallers eyes closed	Fallers eyes closed
*Variable *				
Total path length (cm)				
Before training	198.67 ± 75.21	171.06 ± 32.75	458.20 ± 124.89	383.76 ± 105.29
After training	183.49 ± 57.05	149.52 ± 28.05	403.92 ± 135.64	386.07 ± 110.42
Mediolateral path (cm)				
Before training	137.35 ± 49.43	116.71 ± 22.52	302.16 ± 80.85	259.94 ± 78.3
After training	124.44 ± 29.74	100.87 ± 16.38	259.31 ± 77.89	253.08 ± 80.54
Anteroposterior path (cm)				
Before training	114.35 ± 53.15	100.61 ± 22.96	281.87 ± 85.54	227.84 ± 63.31
After training	108.60 ± 45.03	89.24 ± 23.21	253 ± 93.55	238.59 ± 63.6
Sway area (cm^2^)				
Before training	16.97 ± 5.70	15.79 ± 5.28	66.05 ± 16.20	44.04 ± 16.39
After training	14.70 ± 4.38	10.81 ± 2.98	45.37 ± 15.13	41.48 ± 14.82

## References

[1] Lajoie Y, Gallagher SP (2004). Predicting falls within the elderly community: comparison of postural sway, reaction time, the Berg balance scale and the Activities-specific Balance Confidence (ABC) scale for comparing fallers and non-fallers. *Archives of Gerontology and Geriatrics*.

[2] Berg WP, Alessio HM, Mills EM, Tong C (1997). Circumstances and consequences of falls in independent community-dwelling older adults. *Age and Ageing*.

[3] Zijlstra A, Ufkes T, Skelton DA, Lundin-Olsson L, Zijlstra W (2008). Do dual tasks have an added value over single tasks for balance assessment in fall prevention programs? A mini-review. *Gerontology*.

[4] (2010). IVZ: Hospital services given for injuries and poisonings. *Health Statistic Yearbook*.

[5] Huntzinger A (2011). AGS releases guideline for prevention of falls in older persons. *American Family Physician*.

[6] Tinetti ME (2003). Preventing falls in elderly persons. *New England Journal of Medicine*.

[7] Howe TE, Rochester L, Jackson A, Banks PM, Blair VA (2007). Exercise for improving balance in older people. *Cochrane Database of Systematic Reviews*.

[8] Shubert TE (2011). Evidence-based exercise prescription for balance and falls prevention: a current review of the literature. *Journal of Geriatric Physical Therapy*.

[9] Rugelj D (2010). The effect of functional balance training in frail nursing home residents. *Archives of Gerontology and Geriatrics*.

[10] Granacher U, Gruber M, Gollhofer A (2009). The impact of sensorimotor training on postural control in elderly men. *Deutsche Zeitschrift fur Sportmedizin*.

[11] Halvarsson A, Olsson E, Farén E, Pettersson A, Ståhle A (2011). Effects of new, individually adjusted, progressive balance group training for elderly people with fear of falling and tend to fall: a randomized controlled trial. *Clinical Rehabilitation*.

[12] Rugelj D, Tomšič M, Sevšek F (2012). Effectiveness of multi-component balance specific training on active community-dwelling elderly. *HealthMed*.

[13] Dite W, Temple VA (2002). A clinical test of stepping and change of direction to identify multiple falling older adults. *Archives of Physical Medicine and Rehabilitation*.

[14] Shumway-Cook A, Brauer S, Woollacott M (2000). Predicting the probability for falls in community-dwelling older adults using the timed up and go test. *Physical Therapy*.

[15] Melzer I, Benjuya N, Kaplanski J (2004). Postural stability in the elderly: a comparison between fallers and non-fallers. *Age and Ageing*.

[16] Kelsey JL, Procter-Gray E, Berry SD (2012). Reevaluating the implications of recurrent falls in older adults: location changes the inference. *Journal of the American Geriatrics Society*.

[17] Chamberlin ME, Fulwider BD, Sanders SL, Medeiros JM (2005). Does fear of falling influence spatial and temporal gait parameters in elderly persons beyond changes associated with normal aging?. *Journals of Gerontology A*.

[18] Toulotte C, Thevenon A, Watelain E, Fabre C (2006). Identification of healthy elderly fallers and non-fallers by gait analysis under dual-task conditions. *Clinical Rehabilitation*.

[19] Lord SR, Clark RD, Webster IW (1991). Physiological factors associated with falls in an elderly population. *Journal of the American Geriatrics Society*.

[20] Berg K, Wood-Dauphinee S, Williams JI, Gayton D (1989). Measuring balance in the elderly: preliminary development of an instrument. *Physiotherapy Canada*.

[21] Berg KO, Wood-Dauphinee SL, Williams JI, Maki B (1992). Measuring balance in the elderly: validation of an instrument. *Canadian Journal of Public Health*.

[22] Buchman AS, Boyle PA, Wilson RS, Bienias JL, Bennett DA (2007). Physical activity and motor decline in older persons. *Muscle and Nerve*.

[23] Steffen TM, Hacker TA, Mollinger L (2002). Age- and gender-related test performance in community-dwelling elderly people: six-minute walk test, berg balance scale, timed up & go test, and gait speeds. *Physical Therapy*.

[24] Shumway-Cook A, Horak FB (1986). Assessing the influence of sensory interaction on balance. Suggestion from the field. *Physical Therapy*.

[25] Sevšek F Stabilometrija V 1.0. Ljubljana: Visoka šola za zdravstvo. http://manus.zf.uni-lj.si/~sevsekf/Programi/Stabilometrija.

[26] Rugelj D, Sevšek F (2011). The effect of load mass and its placement on postural sway. *Applied Ergonomics*.

[27] Faul F, Erdfelder E, Lang A-G, Buchner A (2007). G*Power 3: a flexible statistical power analysis program for the social, behavioral, and biomedical sciences. *Behavior Research Methods*.

[28] Melvill Jones G, Kandel ER, Schwartz JH, Jessell TM (2008). Posture. *Principles of Neural Science*.

[29] Wu G, Chiang J-H (1997). The significance of somatosensory stimulations to the human foot in the control of postural reflexes. *Experimental Brain Research*.

[30] Horak FB, Hlavacka F (2001). Somatosensory loss increases vestibulospinal sensitivity. *Journal of Neurophysiology*.

[31] Westlake KP, Culham EG (2007). Sensory-specific balance training in older adults: effect on proprioceptive reintegration and cognitive demands. *Physical Therapy*.

[32] Horak FB, Mirka A, Shupert L, Wollacott MH, Shumway-Cook A (1989). The role of peripheral vestibular disorders in postural dyscontrol in the elderly. *The Development of Posture and Gait: Across the Lifespan*.

[33] Gillespie LD, Robertson MC, Gillespie WJ (2009). Interventions for preventing falls in older people living in the community. *Cochrane Database of Systematic Reviews*.

[34] Melzer I, Kurz I, Oddsson LIE (2010). A retrospective analysis of balance control parameters in elderly fallers and non-fallers. *Clinical Biomechanics*.

[35] Lord SR, Ward JA, Williams P, Anstey KJ (1994). Physiological factors associated with falls in older community-dwelling women. *Journal of the American Geriatrics Society*.

[36] Weerdesteyn V, Rijken H, Geurts ACH, Smits-Engelsman BCM, Mulder T, Duysens J (2006). A five-week exercise program can reduce falls and improve obstacle avoidance in the elderly. *Gerontology*.

[37] Clary S, Barnes C, Bemben D, Knehans A, Bemben M (2006). Effects of ballates, step aerobics, and walking on balance in women aged 50-75 years. *Journal of Sports Science and Medicine*.

[38] Goble DJ, Coxon JP, Wenderoth N, Van Impe A, Swinnen SP (2009). Proprioceptive sensibility in the elderly: degeneration, functional consequences and plastic-adaptive processes. *Neuroscience and Biobehavioral Reviews*.

[39] Waddington GS, Adams RD (2004). The effect of a 5-week wobble-board exercise intervention on ability to discriminate different degrees of ankle inversion, before and after wearing the shoes: a study in healthy elderly. *Journal of the American Geriatrics Society*.

[40] Schilling BK, Falvo MJ, Karlage RE, Weiss LW, Lohnes CA, Chiu LZF (2009). Effects of unstable surface training on measures of balance in older adults. *Journal of Strength and Conditioning Research*.

[41] Rosengren KS, McAuley E, Mihalko SL (1998). Gait adjustments in older adults: activity and efficacy influences. *Psychology and Aging*.

[42] American College of Sports Medicine (2000). *ACSMS's Guidelines for Exercise Testing and Prescription*.

[43] Halsband U, Lange RK (2006). Motor learning in man: a review of functional and clinical studies. *Journal of Physiology Paris*.

[44] Faugloire E, Bardy BG, Stoffregen TA (2006). Dynamics of learning new postural patterns: influence on preexisting spontaneous behaviors. *Journal of Motor Behavior*.

